# Super-Resolved Dynamic 3D Reconstruction of the Vocal Tract during Natural Speech

**DOI:** 10.3390/jimaging9100233

**Published:** 2023-10-20

**Authors:** Karyna Isaieva, Freddy Odille, Yves Laprie, Guillaume Drouot, Jacques Felblinger, Pierre-André Vuissoz

**Affiliations:** 1 IADI, Université de Lorraine, U1254 INSERM, F-54000 Nancy, France; freddy.odille@inserm.fr (F.O.); pa.vuissoz@chru-nancy.fr (P.-A.V.); 2 CIC-IT 1433, CHRU de Nancy, INSERM, Université de Lorraine, F-54000 Nancy, France; 3 LORIA, Université de Lorraine, CNRS, INRIA, F-54000 Nancy, France

**Keywords:** magnetic resonance imaging, super-resolution, vocal tract, dynamic MRI, speech

## Abstract

MRI is the gold standard modality for speech imaging. However, it remains relatively slow, which complicates imaging of fast movements. Thus, an MRI of the vocal tract is often performed in 2D. While 3D MRI provides more information, the quality of such images is often insufficient. The goal of this study was to test the applicability of super-resolution algorithms for dynamic vocal tract MRI. In total, 25 sagittal slices of 8 mm with an in-plane resolution of 1.6 × 1.6 mm^2^ were acquired consecutively using a highly-undersampled radial 2D FLASH sequence. The volunteers were reading a text in French with two different protocols. The slices were aligned using the simultaneously recorded sound. The super-resolution strategy was used to reconstruct 1.6 × 1.6 × 1.6 mm^3^ isotropic volumes. The resulting images were less sharp than the native 2D images but demonstrated a higher signal-to-noise ratio. It was also shown that the super-resolution allows for eliminating inconsistencies leading to regular transitions between the slices. Additionally, it was demonstrated that using visual stimuli and shorter text fragments improves the inter-slice consistency and the super-resolved image sharpness. Therefore, with a correct speech task choice, the proposed method allows for the reconstruction of high-quality dynamic 3D volumes of the vocal tract during natural speech.

## 1. Introduction

MRI has become the gold standard modality for speech imaging [1]. It demonstrates numerous advantages over other imaging techniques such as electromagnetic articulography [2], X-rays [3], and ultrasound imaging [4,5]. However, due to physical constraints, it remains relatively slow, which complicates imaging of fast movements and leads to the presence of obstructing motion artifacts. For this reason, an MRI of the vocal tract is often performed in 2D in mid-sagittal orientation. Multiple techniques allowing for high-quality 2D speech imaging have been developed. Classic cine-MRI, analogous to that used for cardiac imaging, requires several repetitions of the same movement, with different data segments acquired during each repetition. This method was successfully applied to the speech MRI [6,7]. Another approach often called “real-time MRI” allows for high spatiotemporal resolution speech imaging from a single repetition. The substantial gain in temporal resolution is reached due to two principal factors: ultra-short repetition times (TR) and non-cartesian sampling providing good coverage of the k-space center. The reconstruction methods employed for the real-time MRI vary from gridding combined with temporal sliding window technique [8] to SENSE inversion with low-rank and/or sparsity constraints [9,10,11] and regularized non-linear inversion [12].

Nevertheless, 2D images cannot provide complete information on the vocal tract shape which is important for speech production studies. This is why 2D real-time recordings are usually completed with 3D static vocal tract images [13,14]. During such acquisitions, speakers are asked to artificially keep the same articulators’ position for several seconds. However, this approach has a disadvantage: vocal tract shapes acquired with static 3D MRI are often different from their position during natural speech [15].

Dynamic 3D MRI techniques overcome this issue. Fu et al. [16] applied a reconstruction strategy based on inversion with joint low-rank and spatiotemporal total variation constraints. The method was validated with a small corpus based on American English syllables with a spatial resolution of 2.2 × 2.2 × 5.0 mm^3^ and a nominal temporal resolution of 6 ms (achieved with the sliding window technique). Zhao et al. [17] proposed a reconstruction based on spatial and temporal constraints. They demonstrated that the quality of obtained images is comparable to that of 2D scans. However, its relatively low spatial resolution (2.4 × 2.4 × 5.8 mm^3^) may not allow for correct imaging of fine articulators such as epiglottis. Jin et al. [18] presented a protocol with a higher spatial resolution of 1.9 × 1.9 × 2 mm^3^. However, despite the authors demonstrating the potential for linguistic analysis based on the acquired data, the images presented in the publication are significantly compromised by motion artifacts.

While dynamic 3D imaging remains the most prominent technology for visualizing the vocal tract, 2D multislice imaging may have potential benefits. First of all, it enables higher spatial resolution within an acceptable temporal resolution. It is also better sampled which improves the overall quality. Finally, the acquired data directly contains the high-quality mid-sagittal slice. This could help in creating a deep learning model allowing for 2D to 3D mapping based on mid-sagittal images only. Nevertheless, an obvious disadvantage of the 2D multislice strategy is the need to repeat the corpus multiple times. These repetitions are not always temporally aligned. The data related to different slices should be then synchronized in order to reconstruct a 3D volume. Manual linear piece-wise alignment can be employed [19] but is extremely time-consuming. Zhu et al. [20] proposed a strategy based on dynamic time warping of selected mel-frequency cepstral coefficients of the acoustic recording. Finally, a manifold-learning approach for dynamic 3D vocal tract reconstruction was recently presented in [21]. This method allows for joint multi-slice reconstruction and was validated on a small corpus consisting of several utterances.

The most problematic point of 3D reconstruction from multislice vocal tract acquisition is the articulators’ position reproducibility over the slices. This issue can be managed with both acquisition protocol adjustment and improving the reconstruction. The former is related to the human capacity to repeat the same motion sample and could include smaller utterance duration or keeping the same speech rate. Indeed, one can intuitively assume that slower speech rates allow for greater openings. The ability to concentrate so as to memorize the previous manner to pronounce the text plus the absence of misreading also play an important role.

Regarding the reconstruction, interpolation is the most common volume reconstruction method. However, it does not allow for the resolution of details of a size inferior to the slice thickness and fails in cases of inconsistencies between the slices. Both defaults can be overcome with the regularized super-resolution strategy [22]. The method uses redundancy in information from overlapping slices. The slice placement can be designed in three different ways: (1) applying small slice shifts in the slice direction, (2) acquiring three orthogonal volumes, or (3) acquiring oblique slices intersecting between them. The method allows for nearly isotropic 3D volume reconstruction. Imposing spatial smoothness (Tikhonov regularization) helps with the inconsistencies handling but may decrease the sharpness. Delbany et al. [23] demonstrated that Beltrami regularization, which is a form of total variation providing reduced stair-casing effects, improves the sharpness of super-resolved breast MRI.

The goal of this study was to test the applicability of the super-resolution strategy for dynamic vocal tract MRI. We aimed to reconstruct an isotropic volume of 1.6 × 1.6 × 1.6 mm^3^ with a temporal resolution of 20 ms. Contrary to other studies devoted to 3D or pseudo-3D dynamic vocal tract MRI, we focus on good coverage of intra-speaker variability which requires a relatively large speech corpus. In this work, we describe a pre-processing pipeline that allows for reducing the inter-slice inconsistencies before applying the super-resolution strategy. We present the results of applying the super-resolution technique with two different regularizers and compare them with the gold-standard 2D dynamic MRI. Additionally, we estimate the impact of the experimental protocol on the articulators’ position reproducibility.

## 2. Materials and Methods

### 2.1. Volunteers and Speech Task

The volunteers were one male (denoted as S1, 26 years old) and one female (denoted as S2, 33 years old) native French speakers. The data were recorded under the approved ethical protocol “EDEN” (ClinicalTrials.gov Identifier: NCT05218460). This study was approved by the institutional ethics review board (CPP SUD-EST IV, 26.07.21). The acquisition duration was limited by the ethical protocol to 1 h 30 min.

The speakers were asked to read the text “La bise et le soleil…” [24] which demonstrates a good coverage of French phonetic context. For S2, the text has been divided into eight fragments. The text fragments had a duration of 6 to 11 s each and were interleaving (the last word from a fragment was read once again in the next fragment). The fragments can be found in Table S1.

### 2.2. Data Acquisition

The MRI data were recorded at Nancy Central Regional University Hospital on a Siemens Prisma 3T scanner (Siemens, Erlangen, Germany). The speakers were in the supine position and the Siemens Head/Neck 64 coil was used.

The real-time MRI sequence was a radial undersampled 2D FLASH sequence [25]. The repetition time TR was 2.22 ms and the echo time TE was 1.47 ms. The flip angle was 5°. The acquisition of 9 radial spokes allowed for reconstruction of an image with a matrix size of 136 × 136 with an in-plane resolution of 1.6 × 1.6 mm. The field of view was 220 × 220 mm^2^ and the slice thickness was 8 mm. The images were recorded at a frame rate of 50 frames per second and reconstructed with a nonlinear inverse technique presented in [25]. The first 100 images of each acquisition were pre-scans (which were not reconstructed) for the reconstruction initialization.

**Figure 1:**
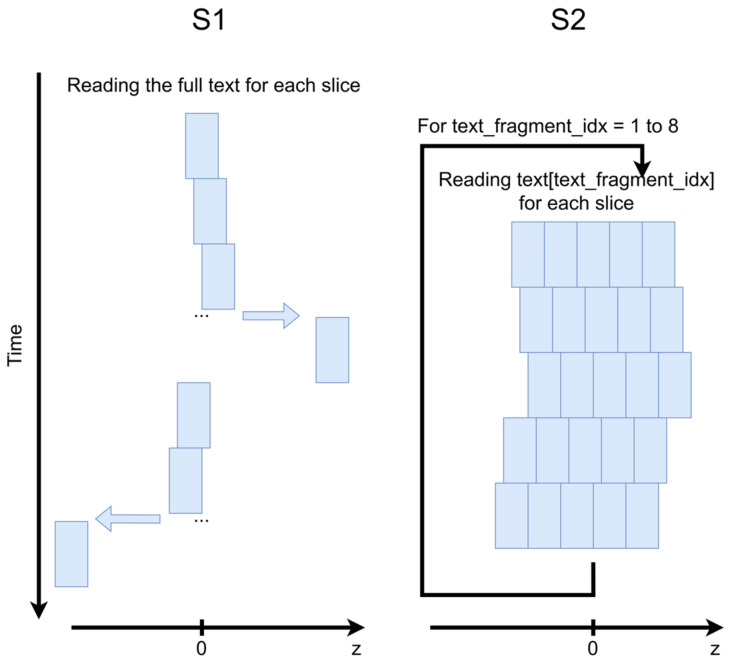
Schematic illustration of the acquisition strategies employed for subjects S1 and S2. The blue rectangles denote slices.

In order to estimate the impact of the acquisition protocol on speech reproducibility, two different strategies were tested (schematically illustrated in Figure 1).

1. S1 was reading the integrality of the text repeatedly 25 times. Each repetition covered one slice and lasted approximately 1 min. The acquisition started from the mid-sagittal slice, then all slices on the right from the mid-sagittal slice were acquired, and finally all slices on the left were acquired. The slice placement order was central to lateral for both sides and the shift between the slices was 1.6 mm. The total acquisition duration was 1 h 20 min and 4000 images were acquired for each slice.
2. S2 was reading 25 repetitions of a small text fragment and then passed to the next one. The repetitions were divided into 5 groups of 5 slices. Each slice group represented a volume of 5 slices (without any distance between them) which were acquired one after one without temporal breaks. The slice groups had a shift of 1.6 mm in the slice direction between them. S2 had a teleprompting visual support forcing her to keep a similar speech rate during each repetition. The teleprompter was launched manually with sequence start. It should be noted that the acquisition of each data slice required 100 pre-scans. Thus, the acquisition protocol used for S2 introduced a longer dead time between acquisitions. Therefore, only fragments 1 to 5 (which correspond to approximately 60% of the text) could be read by S2 due to the ethical protocol restrictions. In total, 1627 images were acquired for each slice.

The sound was acquired simultaneously with the images at a sampling frequency of 16 kHz inside the MRI scanner by using a FOMRI III optoacoustics fiber-optic microphone (FOMRI III, Optoacoustics Ltd., Mazor, Israel). The audio data were recorded by the homemade Signal Analyzer and Event Controller (SAEC) system that was previously designed and validated [26]. A clear speech sample was recorded at the end of each experiment in the same speaker position. The SAEC system also allowed for the recording of transistor-transistor logic (TTL) signals which were emitted by the MRI system at the beginning and at the end of the sequence, and at the beginning and at the end of each slice, for the sound-image synchronization.

### 2.3. Pre-Processing

The super-resolution method cannot be applied directly to the recorded data due to the poor reproducibility of (1) the delay between the sequence start and (2) the speech rate between the repetitions. Therefore, multi-slice volume at each time point should be first formed by temporarily and spatially aligning the slices. In our work, we based the temporal alignment on sound recordings. We also applied rigid in-plane alignment of the images.

**Figure 2:**
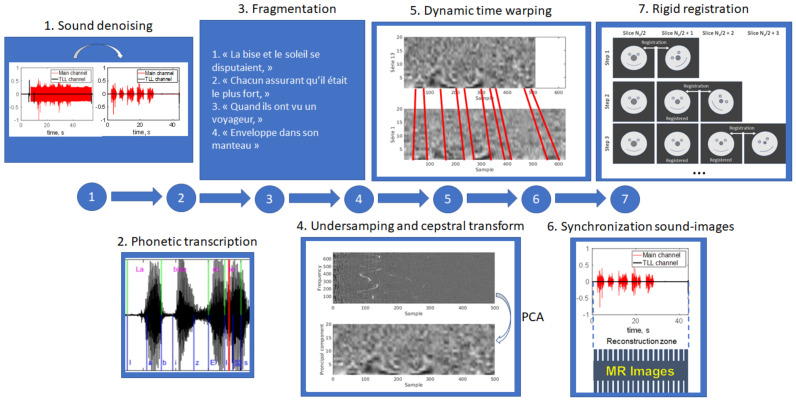
Schematic illustration of the pre-processing pipeline.

The whole proposed pre-processing pipeline included seven steps illustrated in Figure 2.

**Figure 3:**
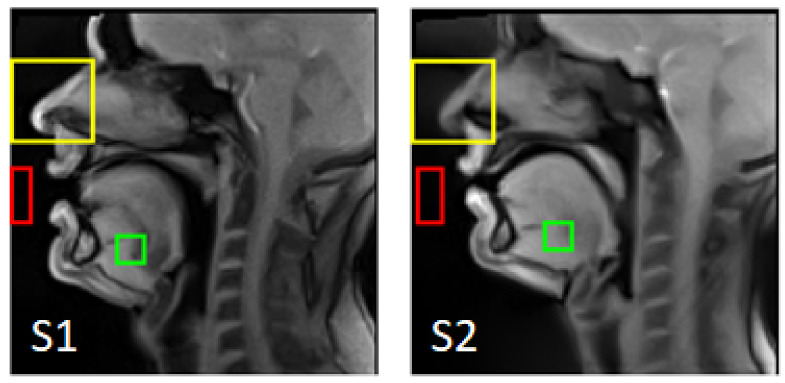
The ROIs used for the rigid registration (yellow), as the background region (red), and as the foreground region (green).

1. Sound denoising. In order to suppress the MRI acoustic noise, we used a source-separation algorithm described in [27]. The Gaussian-mixture model was trained on the speech sample with MRI noise and on the clear speech sample recorded at the end of the protocol.
2. Phonetic transcription (applied only to S1). The text and the audio signal were synchronized by a forced alignment automatic French speech recognition system Astali (https://astali.loria.fr/en/ accessed on 8 September 2023).
3. Text fragmentation. In order to simplify further processing, the text and the corresponding audio recordings of S1 were manually divided on smaller parts which had punctuation or logical pauses between them (“La bise et le soleil se disputaient”, “chaqun assurant qu’il était le plus fort”, “alors qu’ils ont vu on voyageur”, etc.). The resulting fragments are presented in Table S1. The audio recordings corresponding to each volume of S2 were automatically divided into five parts, with each belonging to each separate slice. This was achieved using the TTL signals emitted at the beginning and at the end of each slice and recorded by the SAEC system.
4. Sound feature extraction. We adopted a strategy similar to that proposed in [20]. Firstly, a MATLAB implementation of cepstral transform [28] was applied to the sound recordings. The cepstrum was then 64-times undersampled to facilitate further processing. Oppositely to Zhu et al. [20] who proposed to keep only some fixed cepstrum frequencies, we selected to reduce the feature number by applying the principal component analysis (PCA). The number of principal components to keep was selected to be 20 based on audial and visual comparison of the synchronization quality.
5. Dynamic time warping (DTW). Sound recordings of lateral slices were aligned to that of the mid-sagittal slice using dynamic time warping implementation dtw from the MATLAB Signal Processing Toolbox. The DTW algorithm was applied to the undersampled cepstrum. The fully sampled recordings were aligned using a piece-wise approach: the pieces were warped and the sound samples within each piece remained unchanged.
6. Image-sound alignment. The alignment was performed based on TTL signals recorded by the SAEC system, taking into account the DTW applied to the lateral slices.
7. Rigid registration. Considering the long acquisition time, one can suppose the presence of involuntary head motion. The out-of-plane motion cannot be corrected within the selected multi-slice strategy; however, the in-plane motion can be handled. A region of interest (ROI) including only the subject’s nose (which does not move during speech) was manually selected on the first mid-sagittal image for each subject (see Figure 3). Each lateral slice was registered to the adjacent slice located closer to the center by its translation and rotation:

*For slice* = 1 *to* (*N* − 1)/2*Right slices:*$t_{x},t_{y},\phi =argmax\left\{corr\left[(TR\left(t_{x},t_{y},\phi \right)I_{slice},I_{slice-1}\right]\right\}$ $I_{slice}≔TR\left(t_{x},t_{y},\phi \right)I_{slice}$,*Left slices:*$t_{x},t_{y},\phi =argmax\left\{corr\left[(TR\left(t_{x},t_{y},\phi \right)I_{-slice},I_{-(slice-1)}\right]\right\}$, $I_{-slice}≔TR\left(t_{x},t_{y},\phi \right)I_{-slice}$,*end*where, $t_{x},t_{y},\phi$ are global translation and rotation values, $\hat{T}\left(t_{x},t_{y}\right)$ and $\hat{R}\left(\phi \right)$ are corresponding translation and rotation operators, and $I_{slice}$ are pixel values inside the selected ROI. slice>0 corresponds to the right slices, slice<0 corresponds to the left slices, and slice=0 corresponds to the mid-sagittal slice. The cross-correlation maximization was performed by using the *fminunc* function from the MATLAB Optimization Toolbox, and the interpolation after registration was performed using the MATLAB *interp2* function.

### 2.4. Super-Resolution

The super-resolved images of 1.6 × 1.6 × 1.6 mm^3^ were obtained by solving the following inverse problem [23]:

$$x=\underset{x}{\mathrm{argmin}}{\sum_{i=1}^{5}\left\|D_{i}B_{i}M_{i}x-\rho_{i}\right\|}^{2}+\lambda C(x),$$

where i—is the acquired volume index, x—is the super-resolved volume, ρ—acquired anisotropic images, D—downsampling operator, B—blurring operator (calculating mean within a rectangular volume), M—geometrical transform (shift in slice direction). We tested two different regularizers: $C\left(x\right)={\left\|x\right\|}^{2}$ with $\lambda =10^{-6}$—Tikhonov regularization, and $C\left(x\right)={\left(1+{\beta^{2}\left|\nabla x\right|}^{2}\right)}^{1/2}$ with β=1 and $\lambda ={5\cdot 10}^{-5}$—Beltrami regularization. The parameter values were selected after a test on a small amount of the vocal tract images (50-th image of each text fragment). The smallest values which did not generate reconstruction artifacts were selected. The super-resolution reconstruction code was previously developed in a MATLAB environment and validated on cardiac and breast MRI applications [23,29]. The optimization problem was solved using the conjugated gradient descend method for Tikhonov regularization and with the primal-dual projected gradient solver [30] for Beltrami regularization. We set the convergence tolerance to 10^−3^ and the maximum number of iterations to 256.

### 2.5. Validation

All calculations were performed on a workstation with Intel(R) Xeon(R) with 28 cores of 2.5 GHz and 64 GB RAM.

The sound synchronization was evaluated manually in two ways: visually, by comparing the cepstrograms and the synchronized audio recordings, and audially, by superimposing the recordings. Then, the image synchronization and quality of the rigid registration of the final super-resolved images were evaluated visually using 3D dynamic videos in different projections. The 3D volumes were rendered using the volshow function from the MATLAB Image Processing Toolbox in iso-surface mode with an automatically selected threshold.

To evaluate the final super-resolved image quality, we used the sharpness index [23] and signal-to-noise ratio (SNR) as quantitative metrics. The SNR was estimated from two ROIs (a foreground region $I_{fg}$ and a background region $I_{bg}$) per its definition $SNR=mean(I_{fg})/std(I_{bg})$. The regions were selected so that the foreground contains high signal tissues and the background does not contain any tissue during the volunteer’s articulation. The selected regions are presented in Figure 3.

Both metrics were applied to compare the super-resolved images with the aligned registered 2D images and to compare different regularization approaches between them. The whole image stack of each volunteer (concatenated for all fragments) was used for the analysis. A paired *t*-test was employed for statistical analysis of the sharpness metrics.

To estimate the smoothness in the slice direction, in-plane regions of interest were first defined for each volume. They were selected to be the edges detected on the mid-sagittal images with Canny edge detector (from MATLAB Image Processing Toolbox with default parameters). Indeed, the most pronounced inter-slice inconsistencies may appear close to boundaries, where intensity rapidly changes. Then, for each edge pixel, curves in the slice direction (left-right direction) were extracted, so that each curve consists of 25 points. The curves were fitted using MATLAB smoothing splines (Curve Fitting Toolbox) with default parameters. The smoothness metric was defined as follows:

$$S=\sqrt{\frac{\sum_{slice}{\left({IF}_{slice}-I_{slice}\right)}^{2}}{\sum_{slice}{I_{slice}}^{2}}}$$

Here, $I_{slice}$ is the pixel value and ${IF}_{slice}$ is the value of the fitting curve. The metric was calculated independently for all edge points and for all volumes.

## 3. Results

### 3.1. Pre-Processing

**Figure 4:**
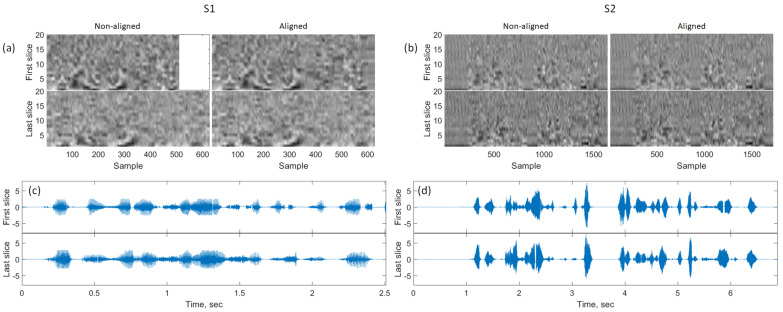
Illustration of the sound alignment quality. (**a**,**b**): First 20 principal components of the cepstrum for S1 and S2, correspondingly. (**c**,**d**): Examples of sound recordings after the dynamic time warping. Note the white spaces are present in a few places due to the piece-wise alignment.

Audial evaluation of superimposed speech recordings after DTW and visual evaluation of their plots have shown a good sound alignment quality. Examples of aligned sound recordings and their cepstrograms (20 principal components) are presented in Figure 4 and the superimposed audio recordings can be found in Supplementary Materials: Audio S1 for the recordings before the alignment, and Audio S2 for the aligned audio recordings. One can, however, notice a slight difference in sound intensity across the times indicating imperfections in inter-slice reproducibility. It is also important to note that in the case of S1, the speech rate and the acquisition duration might be substantially different for different slices (see Figure 4a). The cepstrum patterns of fixed text fragments also varied more for S1 than for S2.

**Figure 5:**
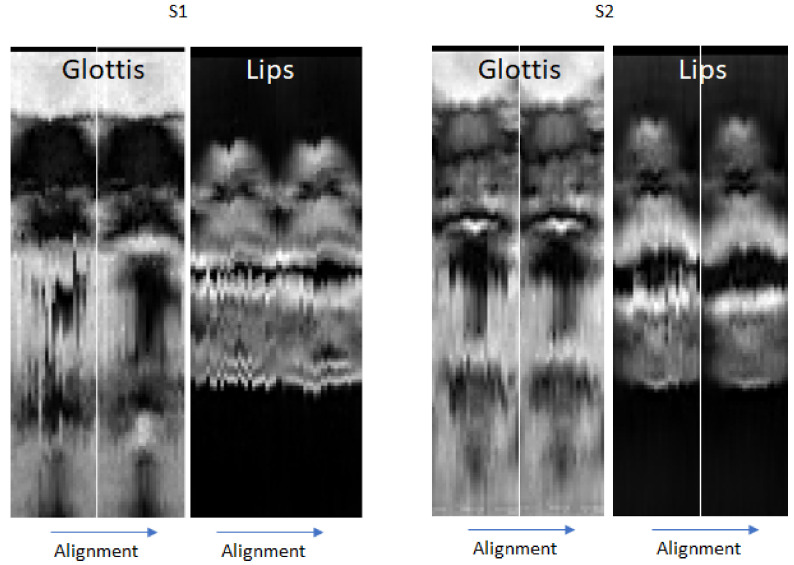
Examples of visualization of the alignment quality in two different coronal planes: the glottis region (on the left for each subject) and the lips region (on the right for each subject) for S1 and S2. The non-aligned slices are on the left of each pair and the aligned ones are on the right of each pair.

Some inter-slice inconsistencies were still present after the temporal sound-based alignment. Visual analysis of generated video sequences has shown that these imperfections were related rather to insufficient reproducibility than to temporal inconsistency. Figure 5 demonstrates typical examples of temporally aligned slices during vowel production. One can notice a considerable difference in the glottis position and in the lips opening between the left side and the right side of S1. At the same time, only small inter-slice variations can be observed for S2.

**Figure 6:**
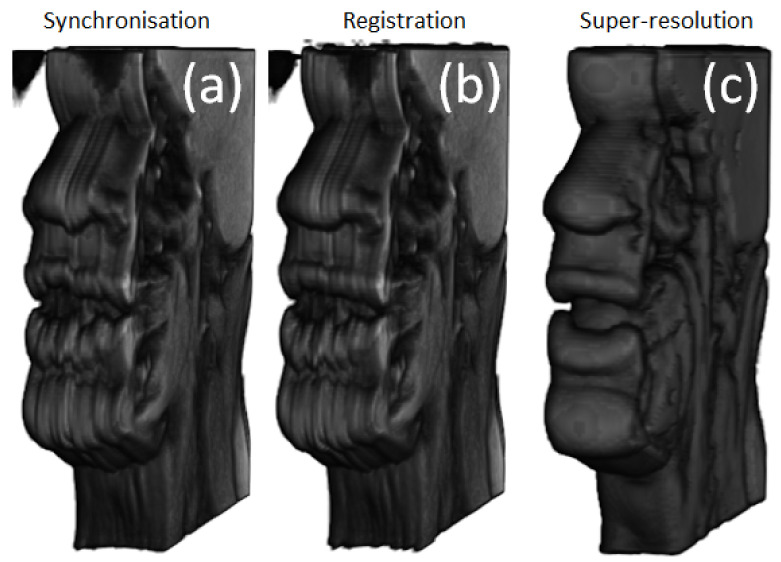
A rendered 3D volume of S1 illustrating different processing steps. (**a**) After temporal alignment only. (**b**) After rigid registration. (**c**) After super-resolution application.

The incoherencies that could be explained by rigid head motion (visible outside of the vocal tract region) were more remarkable on S1 images, mostly on the left side (see Figure 6a). It can be noticed that rigid registration improved the consistency between slices, especially visible on the nose and the upper lip (see Figure 6b). The rigid registration took 5 to 6 s per volume.

### 3.2. Super-Resolution

Calculating a super-resolved volume took 4 to 5 s. The convergence criterion was generally reached after 25 to 30 iterations.

**Figure 7:**
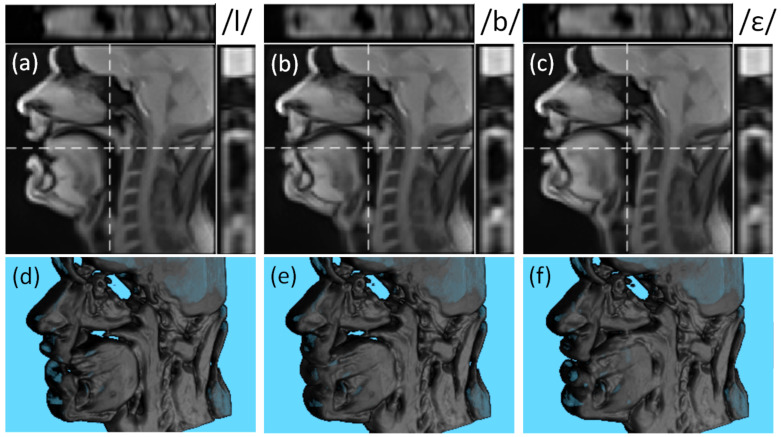
Examples of the reconstructed super-resolved 3D volume of S1 for phonemes/l/ (**a**,**d**), /b/ (**b**,**e**), and /ε/ (**c**,**f**). The subfigures (**a**–**c**) show the mid-sagittal slice, the central axial slice (on the top and denoted as the horizontal dashed line), and the central coronal slice (on the right and denoted as the vertical dashed line). The subfigures (**d**–**f**) demonstrate the rendered super-resolved 3D volumes.

The examples of the reconstructed super-resolved 3D volumes for different phonemes are presented in Figure 7. Despite demonstrating some blur around the articulators, the contours remain well-distinguishable in all three planes.

The visual evaluation of the generated video sequences confirmed the air-tissue boundary was unambiguous in most images. The articulatory positions corresponded well to the pronounced phonemes. The examples of the generated videos are presented in Videos S1 and S2.

**Figure 8:**
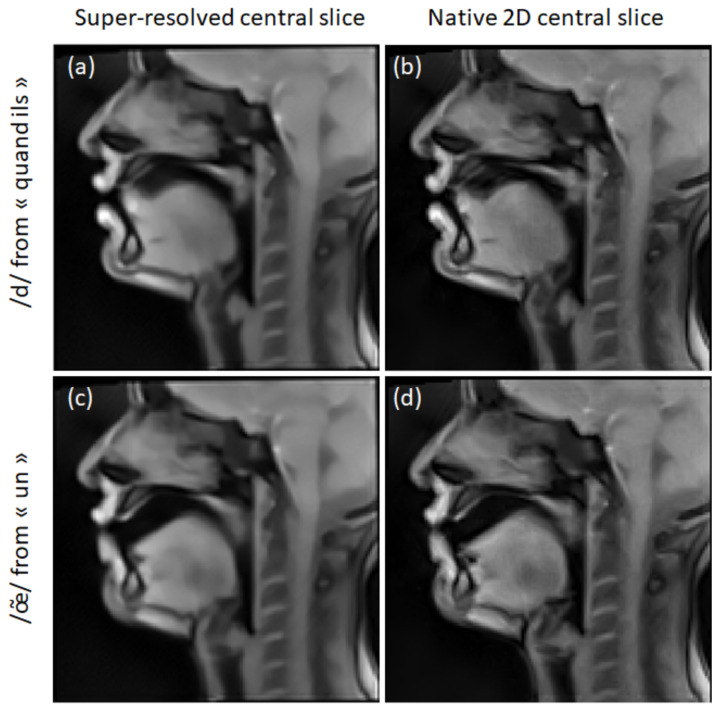
Examples of super-resolved mid-sagittal slices of S2 (**a**,**c**) in comparison to the native 2D mid-sagittal slices (**b**,**d**). The Beltrami regularization was used for the super-resolution.

Figure 8 shows super-resolved and 2D mid-sagittal images of S2. One can notice a texture difference: the super-resolved images are smoother due to edge regularization and denoising. This effect is especially visible in stationary regions, such as the brain. In some cases, super-resolution improved the visualization of some articulators (like the soft palate in Figure 8a,b). In other cases, super-resolution decreased the region’s sharpness (like the tongue tip in Figure 8c,d).

**Figure 9:**
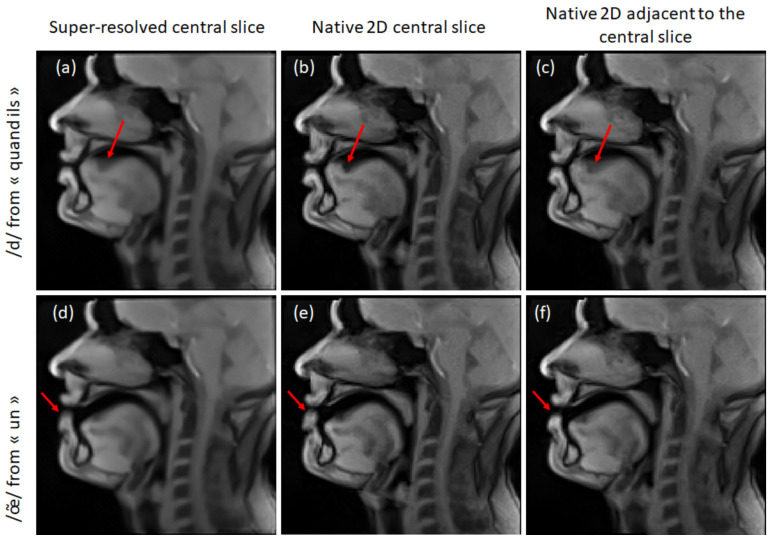
Examples of the super-resolution failure for S1: super-resolved mid-sagittal slices (**a**,**d**), native 2D mid-sagittal slices (**b**,**e**), and adjacent to the mid-sagittal slices (**c**,**f**). Beltrami regularization was used for the super-resolution. The red arrows point to the blurry regions discussed in the text.

S1 demonstrated a worse super-resolved image quality. Some typical cases of its failure are presented in Figure 9. It can be seen that the tongue dorsum region is much sharper in 2D images (Figure 9b) than in the super-resolved images (Figure 9a). This can be explained by the migration of a motion artifact from an adjacent slice (Figure 9c). Another typical case is the difference in articulation (poor inter-slice reproducibility). The comparison of two adjacent slices shows a difference in the lips opening (Figure 9b,c). The resulting super-resolved image demonstrates a blur located between the two lips positions.

**Figure 10:**
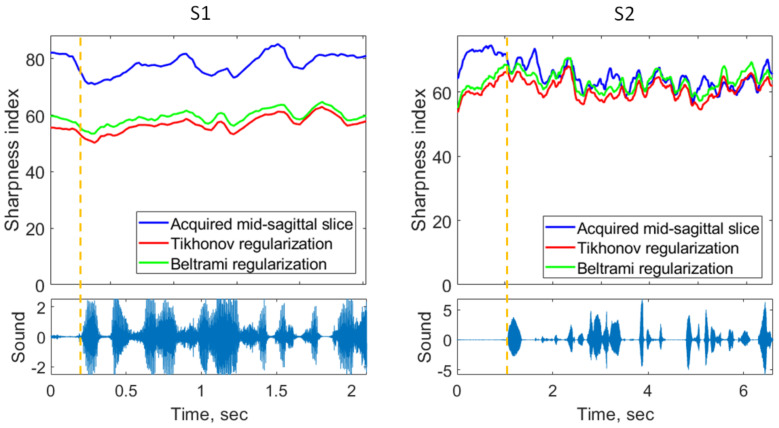
Upper plots: examples of sharpness index change in time for mid-sagittal slices of S1 and S2. Lower plots: corresponding sound recordings. The dashed vertical orange line shows the beginning of the speech.

These visual observations were confirmed with the sharpness index. Examples of the sharpness index of the mid-sagittal images are depicted in Figure 10. One can observe considerably lower sharpness indices for the super-resolved images of S1, for both Tikhonov and Beltrami regularizers. The super-resolved mid-sagittal images of S2 demonstrated, on average, slightly lower sharpness than the native 2D mid-sagittal images. The segment with the best super-resolved image sharpness is presented in Figure 10.

**Figure 11:**
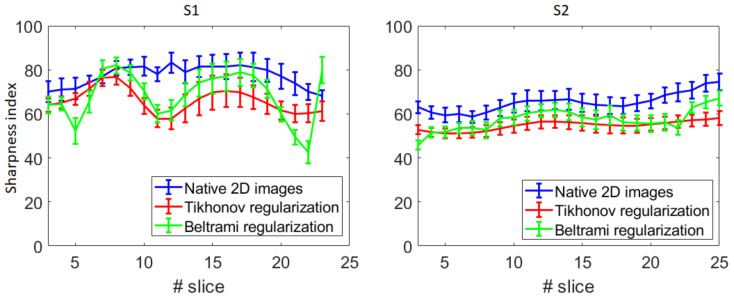
Average and standard deviation of the sharpness index for each slice of S1 and S2.

The sharpness index dependence on the slice is presented in Figure 11. It can be seen that in the case of S1, the images around the mid-sagittal slice demonstrate lower sharpness. In contrast, in the case of S2, the sharpness has an almost uniform distribution.

The difference between the sharpness indices of the native 2D images and the super-resolved images (both regularizers) was highly significant (*p* << 10^−6^) for both subjects, with the super-resolved images being less sharp. The sharpness of the images reconstructed with the Beltrami regularization appeared to be significantly superior to the Tikhonov regularization (with *p* << 10^−6^) for both volunteers.

The mean and standard deviations of the signal-to-noise ratio are provided in Table 1. Oppositely to the sharpness, the signal-to-noise ratio was the highest for the super-resolved images with the Tikhonov regularization, followed by the super-resolved images with the Beltrami regularization. The native 2D images demonstrated the lowest signal-to-noise ratio. All differences were highly significant (*p* << 10^−6^).

Visually, the super-resolved images demonstrated high smoothness between the slices (see Figure 6c). These visual observations were confirmed with the quantitative results.

**Figure 12:**
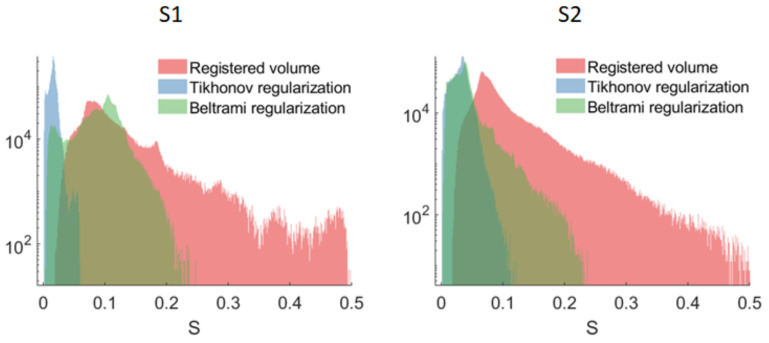
Distributions of the smoothness metric S for the subjects S1 and S2. Note the vertical axis is in logarithmic scale.

**Figure 13:**
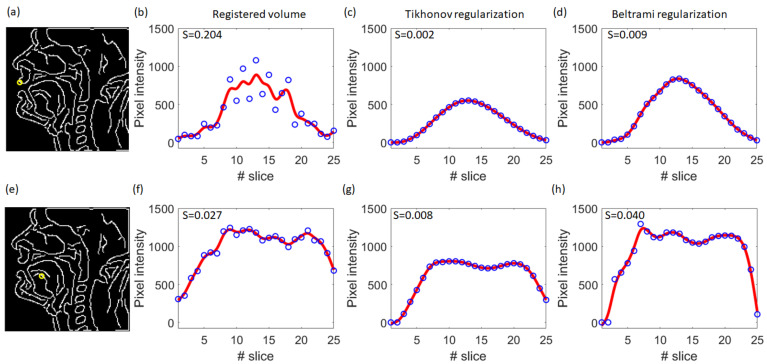
Illustration of the smoothness evaluation steps on highly mobile and moderately mobile regions for S1. (**a**) Results of the Canny edge detection on the mid-sagittal slice image from the super-resolved volume with Tikhonov regularization. The yellow circle indicates the position of a highly mobile region (upper lip) corresponding to the curves (**b**–**d**). (**b**) Pixel values (blue circles) and the smoothing spline fitting curve (red line) for fixed in-plane position for different slices extracted from the registered volume. (**c**) The same as (**b**) extracted from the super-resolved volume with Tikhonov regularization. (**d**) The same as (**b**) extracted from the super-resolved volume with Beltrami regularization. (**e**–**h**): The same for a moderately mobile region in the tongue body. The values in the upper left corner correspond to the smoothness metrics.

Figure 12 shows the distributions of the smoothness metric S for different types of reconstruction. It can be seen that the super-resolved volumes reconstructed with Tikhonov regularization were remarkably smoother than volumes obtained with other reconstruction types. The highest inter-slice inconsistencies were present in volumes reconstructed with rigid registration only. The smoothing effect was more pronounced for the highly mobile parts of the vocal tract: lips, epiglottis, soft palate (see Figure 13a–d). For the less mobile parts, the inter-slice transition was already smooth enough (see Figure 13e–h), so these regions did not substantially contribute to the metric.

### 3.3. Comfort

From the comfort point of view, both volunteers evaluated the selected speech task as tiring (especially, S2). Additionally, the S2 acquisition protocol required adjustment text-sequence synchronization and introduced more dead time which resulted in a longer but more consistent acquisition per fragment.

## 4. Discussion

In this study, we demonstrated the applicability of the super-resolution strategy for dynamic 3D vocal tract reconstruction. We have shown that the super-resolved images are smooth, correspond to the pronounced phonemes, and demonstrate unambiguous air-tissue boundaries in most cases. The resulting isotropic resolution enables the generation of images in any orientation. However, like other existing methods [16,17,18,20,21], the proposed method generates images that contain some residual artifacts related to fast motion and inter-slice inconsistencies. Its direct comparison with the already published techniques is not straightforward due to several reasons. First of all, the proposed technique was evaluated with a challenging speech task. In contrast to some published research, which focused on pronouncing only syllables or isolated words, our approach involved continuous natural speech. The second reason is a higher spatial resolution (1.6 × 1.6 × 1.6 mm^3^ in our case compared to 1.9 × 1.9 × 2 mm^2^ to 2.4 × 2.4 × 5.8 mm^3^ in other studies). Finally, the utilization of different acquisition and reconstruction techniques changes the image appearance (signal-to-noise ratio, contrast, and type of aliasing artifacts). Therefore, only subjective visual comparison with other methods can be performed. We compared our study to only those who published video sequences. The proposed method possibly underperformed the method presented by Zhao et al. [17] in terms of both quality and comfort. However, despite a significantly longer acquisition time, the super-resolved images demonstrated considerably fewer motion artifacts than the 3D dynamic images presented in [18]. Regarding the techniques employing multi-slice alignment, the temporal alignment quality was similar to that presented in [20]. Our protocol implies a higher spatial resolution and allows for the generation of smoother images at the cost of larger acquisition time and the presence of inconsistencies not related to the alignment (in the case of S1). Finally, the quality of the resulting volume was comparable to that presented in [21] in terms of artifacts. Thus, the proposed method can be considered as a working approach for 3D volume reconstruction.

It is important to note that, contrary to other multi-slice methods, the super-resolution allows for a guaranteed smooth transition between the slices. It could be helpful in the case of 3D aero-acoustic simulations where all irregularities may generate distortions. Despite this smoothing compromising image sharpness, it allows for the reconstruction of an averaged vocal tract shape with a higher signal-to-noise ratio. This approach could be employed for the creation of a vocal tract atlas [19].

We have observed some differences in the quality of the resulting images of different volunteers. In particular, the images were less sharp with more artifacts related to the inter-slice inconsistency. These distinctions might be explained by the natural difference in the volunteers’ speech; however, it is more likely that these differences are related to the acquisition protocol choice. The first evidence that supports this conclusion is the pronounced difference in articulation in the left and the right slices of S1 (see Figure 4 and Figure 5). These variations can be perfectly explained by the acquisition order (see Figure 1 for the reminder). The temporal difference between the central and the adjacent on the left slice was on the order of 30 min. This delay might affect S1’s speech properties. Another sign that points to the protocol’s importance is the distribution of the sharpness index across different slice positions. The central slices of S1, which are the most sensitive to articulators’ positions, demonstrated a sharpness much lower than that of the native 2D images. The gap between the super-resolved and the native 2D images of S2 was, on the contrary, almost constant over the slices. Finally, the initial 2D images of S1 contained more motion artifacts which migrated into the super-resolved images. These artifacts could be due to the higher speech rate in the absence of visual stimuli.

The overall sharpness was significantly lower than that of the native 2D images. This degradation is probably related to some inter-slice inconsistencies. Nevertheless, it can also be partially explained by the denoising. Indeed, the super-resolution algorithms are also known for their denoising properties due to spatial regularization [23]. The denoising may improve the segmentation potential of the images. By comparing two regularization approaches, we demonstrated that the Beltrami regularization allows for a superior sharpness. These results are consistent with the previous results on the super-resolved breast diffusion MRI [23].

The small participant number is a limitation of this study. Due to the comfort issue, we decided to limit the number of participants for now. Nevertheless, we acquired a relatively large corpus with more than 1500 super-resolved images reconstructed for each subject. This data quantity allows for further development of individual 3D segmentation and 2D to 3D mapping models. Indeed, despite its low sharpness, the air-tissue boundary for both volunteers was unambiguous and potentially allowed for its automatic segmentation. 2D to 3D mapping is also potentially possible, which would allow for the extension of the existing corpus by acquiring only mid-sagittal images. Further work will include exploring these avenues and the acquisition of a larger dataset in case of favorable outcomes in these directions.

## 5. Conclusions

The proposed algorithm allows for a dynamic 3D super-resolved vocal tract reconstruction during natural speech. While time-consuming, the proposed approach offers 3D volumes with a sharpness sufficient for potential automatic segmentation.

The Beltrami regularization allowed for achieving a sharpness superior to that of the Tikhonov regularization. We also demonstrated that correct handling of the speech task improves the reproducibility and, consequently, the image quality. Despite being less sharp than the original 2D images, the resulting super-resolved volumes had a higher signal-to-noise ratio and regular transitions between the slices. This enables further application of these 3D images for aero-acoustic simulations.

## Figures and Tables

**Table 1:** Signal-to-noise ratio (mean ± std) estimated from ROIs on images generated with different reconstruction methods.

Method	S1	S2
Native 2D	76.3 ± 10.4	47.1 ± 7.8
Tikhonov	95.0 ± 14.1	58.5 ± 8.1
Beltrami	81.0 ± 7.7	55.7 ± 6.5

## Data Availability

The data presented in this study are available on request from the corresponding author. The data are not publicly available due to ethical restrictions.

## Supplementary Materials

The following supporting information can be downloaded at: https://www.mdpi.com/article/10.3390/jimaging9100233/s1, Table S1: The text fragments used for each volunteer.; Video S1: Example of super-resolved dynamic series of volunteer S1; Video S2: Example of super-resolved dynamic series of volunteer S2; Audio S1: super-imposed raw audio-recordings of S1; Audio S2: super-imposed aligned audio-recordings of S1.
