# Supplementary material for: Super-Resolved Dynamic 3D Reconstruction of the Vocal Tract during Natural Speech
Source: J Imaging. 2023 Oct 20;9(10):233. doi: 10.3390/jimaging9100233 (PMC10607793; doi:10.3390/jimaging9100233)
Supplement: Supplementary file 1 [file jimaging-09-00233-s001.zip › SupplementaryTableS1.pdf]

#	S1	S2
1	**La bise et le soleil se disputaient**	**La bise et le soleil se disputaient, chacun assurant qu'il était le plus fort, quand**
2	**chacun assurant qu'il était le plus fort**	**fort, quand ils ont vu un voyageur qui s'avançait, enveloppé dans son manteau. Ils**
3	**quand ils ont vu un voyageur qui s'avançait**	**manteaux. Ils sont tombés d'accord que celui qui arriverait le premier à faire ôter son**
4	**enveloppé dans son manteau**	**son manteaux au voyageur serait regardé comme le plus fort. Alors**
5	**Ils sont tombés d'accord**	**fort. Alors la bise s'est mise à souffler de toute sa force mais plus elle soufflait, plus**
6	**que celui qui arriverait le premier**	plus le voyageur serrait son manteaux autour de lui et à la fin, la bise a renoncé à le lui faire ôter. Alors
7	**à faire ôter son manteaux au voyageur**	ôter. Alors le soleil a commencé à briller et au bout d'un moment, le voyageur, réchauffé a ôté son manteau.
8	**serait regardé comme le plus fort.**	manteaux. Ainsi, la bise a dû reconnaître que le soleil était le plus fort des deux.
9	**Alors la bise s'est mise à souffler**	
10	**de toute sa force mais plus elle soufflait**	
11	**plus le voyageur serrait son manteaux**	
12	**autour de lui et à la fin,**	
13	**la bise a renoncé à le lui faire ôter.**	
14	**Alors le soleil a commencé à briller**	
15	**et au bout d'un moment, le voyageur,**	
16	**réchauffé a ôté son manteau.**	
17	**Ainsi, la bise a dû reconnaître**	
18	**que le soleil était le plus fort des deux.**	

Supplementary Table S1. The text fragments used for each volunteer. The bold denotes the actually acquired data.
